# Engagement, motivation, or sustained attention? Rethinking the effects of technology in autism

**DOI:** 10.3389/fdgth.2026.1802286

**Published:** 2026-04-17

**Authors:** Anna Meduri, Chiara Marraffa, Gaia Roccaforte, Paola Chilà, Chiara Failla, Giorgio Gugliotta, Giovanni Pioggia, Flavia Marino

**Affiliations:** 1 Institute for Biomedical Research and Innovation (IRIB), National Research Council of Italy (CNR), Messina, Italy; 2 Department of Cognitive, Psychological Science and Cultural Studies, University of Messina, Messina, Italy; 3 Department of Biomedical, Dental and Morphological and Functional Imaging Sciences, University of Messina, Messina, Italy; 4 Faculty of Psychology, International Telematic University Uninettuno, Roma, Italy

**Keywords:** autism spectrum disorder, engagement, outcome measurement, robot-assisted therapy, sustained attention, virtual reality

## Abstract

Technology-based interventions for Autism Spectrum Disorder (ASD) are frequently justified on the grounds that digital tools “increase engagement” and “enhance motivation.” However, across domains such as robot-assisted therapy, immersive environments (virtual and augmented reality), and ICT-based educational applications, outcomes labeled as engagement are often derived from observable indicators including gaze, time-on-task, interaction duration, task adherence, or reduced off-task behavior. While informative, these measures may primarily index sustained attention and, when considered in isolation, do not provide sufficient evidence to support inferences about intentional involvement or intrinsic motivation. In this Perspective paper, we argue that part of the literature implicitly equates increased on-task behavior with increased engagement, despite engagement and motivation being inferential constructs that require clearer operationalization. We first clarify conceptual distinctions between engagement, motivation, and sustained attention, highlighting how overlapping behavioral indicators can lead to interpretative ambiguity. We then summarize a recurring evidence pattern showing that technology-related outcomes are most consistently captured through markers of attentional stability during task performance. Finally, we propose an alternative interpretation: technology may function as a context that supports sustained attention in ASD by leveraging predictable structure, sensory coherence, repetition, and immediate feedback, and in some cases by aligning with restricted interests while the indicators most commonly reported are insufficient to determine whether motivation or deeper forms of engagement have increased. We conclude that improving conceptual precision and measurement practices is essential to interpret intervention outcomes accurately and to identify which technological components modulate attention, motivation, and active participation in autistic individuals.

## 1 Introduction

### 1.1 A widespread claim in technology-based autism interventions

In recent years, the use of technology in interventions for individuals with Autism Spectrum Disorder (ASD) has expanded substantially (1). Tablets, educational apps, social robots, virtual reality environments, and AI-based systems have become increasingly common in clinical and educational settings (2). When practitioners or researchers are asked why these tools are employed, the most frequent explanation is broadly similar: technology “enhances engagement,” “increases motivation,” or “captures interest” more effectively than traditional approaches (3–8). This explanation has become so widespread that it is now often taken for granted. In many cases, these claims are supported mainly by observable indicators such as sustained gaze, time-on-task, and interaction duration.

### 1.2 Conceptual ambiguity in the interpretation of outcomes

However, a closer examination of the literature reveals a considerable conceptual ambiguity. The terms engagement, motivation, and attention are frequently used interchangeably, despite referring to distinct psychological processes. Behaviors such as maintaining eye gaze on a screen, orienting toward a robot, or staying focused on a digital task are commonly interpreted as indicators of strong engagement or high intrinsic motivation. But are we certain that these behaviors reflect genuine engagement? Or might we instead be observing something different: an increase in sustained attention, driven not by the content of the activity but by the intrinsic characteristics of the technological medium? This question becomes particularly relevant in autism, where attentional style and the presence of restricted interests can strongly influence responses to digital stimuli (9).

### 1.3 The present proposal

This manuscript is a conceptual Perspective. Its purpose is to develop an interpretive framework for distinguishing observable indicators from the constructs they are often taken to represent in technology-based ASD interventions, drawing on illustrative examples. Many of the features that make technology appealing predictable structure, repetitiveness, immediate feedback, reduced social ambiguity, and sensory coherence are also those that can naturally amplify attentional focus in autistic individuals (10). From this perspective, the “engagement” often reported in the literature may not result from increased motivation or active participation, but rather from the way technological environments align with the cognitive-attentional profile typical of ASD. In this Perspective article, we propose that part of the literature on technology-based interventions for autism is grounded in an assumption that is rarely made explicit: that an increase in “on-task” behaviors automatically reflects an increase in engagement. To clarify the scope of this argument across modalities, we refer to representative examples from robotics, VR/AR, and ICT interventions, selected because they rely primarily on on-task indicators to support claims about engagement or motivation. Relevant studies were identified through targeted searches in PubMed and Scopus, focusing on English-language publications available up to January 2026 and addressing robotics, virtual/augmented reality, and ICT-based interventions for autistic individuals. The search strategy combined terms related to autism, technology domains, and outcome constructs, with particular attention to studies in which observable behavioral indicators such as gaze, time-on-task, interaction duration, task completion, or reductions in off-task behavior were interpreted as evidence of engagement or motivation. Studies were retained when they examined autistic participants interacting with technological tools and reported behavioral outcomes used to support such claims, whereas purely technical papers, protocol-only reports, and studies lacking relevant behavioral indicators were not considered. Eight illustrative studies are summarized in Supplementary Table S1 to provide a descriptive mapping of recurrent measurement practices across domains. However, on-task behavior is an observable indicator, whereas engagement and motivation are inferential constructs that require clearer operationalization. We argue instead that many observed effects may be more accurately interpreted, at least in part, as variations in sustained attention. This interpretation does not exclude engagement; rather, it specifies what can be inferred when evidence is limited to on-task indicators. Clarifying this distinction is not a marginal theoretical exercise, but a necessary step for identifying which psychological process is actually activated by technology and for interpreting intervention outcomes in a manner that is more precise and consistent with autistic cognitive functioning. To address this issue, it is first necessary to clarify how engagement, motivation, and sustained attention are defined and operationalized within the existing literature. We then specify which indicators most directly support attentional interpretations and what additional evidence is required to substantiate claims about engagement or motivation.

## 2 Clarifying the distinction between engagement, motivation, and sustained attention

In the literature on technology use in autism, the terms engagement, motivation, and attention frequently appear side by side (7, 11, 12). This proximity, however, does not imply that they refer to the same psychological construct. A key issue is that similar behavioral indicators, such as gaze, time-on-task, and task completion, are often used to infer different psychological processes, leading to conceptual overlap and interpretative ambiguity. Clarifying their distinct meanings is essential for understanding which dimension is actually influenced when autistic individuals interact with technological tools. Engagement is commonly described as a multidimensional construct encompassing behavioral participation, emotional involvement, and cognitive investment in an activity (13). An individual may be considered “engaged” when they participate intentionally, respond contingently, and remain mentally oriented toward the goal of the task. Motivation refers to the internal processes intrinsic or extrinsic that initiate and sustain goal-directed behavior. Studies on educational and assistive technologies, for instance, often report increases in “perceived motivation” based on teacher or caregiver impressions, though such reports rarely specify whether this motivation is intrinsic or linked to structural features of the tool itself (10). Sustained attention, in contrast, is a cognitive process involving the ability to maintain focus on a stimulus over time while resisting distraction (14). This process does not necessarily indicate interest or emotional involvement. In several studies on immersive technologies, for example, indicators such as prolonged looking time, reduced distractibility, or continuous task involvement are recorded as signs of “engagement,” although these behaviors may more accurately reflect increased attentional maintenance (15). Despite these distinctions, many empirical studies rely on observable behaviors such as looking toward a robot, orienting to a screen, or completing a digital task as evidence for engagement or motivation. This is particularly evident in work on robot-assisted therapy, where engagement levels are inferred from facial expressions, body orientation, or time spent in interaction (11), measures that can plausibly index attentional processes rather than motivational ones. In educational or home-based ICT contexts, review-level evidence and primary studies suggest a comparable pattern, with participation, engagement, or motivation often inferred from teacher or caregiver reports rather than from standardized instruments (16, 17). Accordingly, time- and gaze-based indicators most directly support attentional interpretations, whereas claims about engagement or motivation require convergent, construct-specific evidence. Engagement inferences should be supported by indicators of active participation and documented affective and cognitive involvement, while motivation inferences require evidence of choice, voluntary re-engagement, and willingness to sustain effort under increasing cost. Highlighting this distinction does not imply that previous interpretations are inaccurate; rather, it emphasizes that a single observable behavior may arise from different psychological mechanisms. An increase in time spent looking at a device, for instance, may indeed reflect genuine engagement, but it may equally be explained by features that naturally support sustained attention such as repetition, visual coherence, predictability, or alignment with restricted interests (18). These conceptual distinctions are particularly relevant when examining how outcomes are measured and interpreted in empirical studies on technology use in autism. To facilitate this conceptual distinction, Table 1 provides an overview of how engagement, motivation, and sustained attention are typically defined and operationalized in technology-based autism research.

**Table 1:** Conceptual distinctions between engagement, motivation, and sustained attention in technology-based autism research. The table summarizes common definitions and indicators used in empirical studies, clarifies what these indicators most directly capture, and specifies additional evidence required to support inferences about engagement and motivation.

Construct	Definition	Typical indicators used in studies	What these indicators primarily capture	Additional evidence needed to support the construct
Engagement	Multidimensional construct involving behavioral, emotional, and cognitive involvement	On-task indicators often used as engagement proxies: time-on-task, interaction duration, gaze, task completion	Observable participation; often inferred involvement	Markers of active participation, such as spontaneous initiation, contingent responding, reciprocity, and strategy use. Structured evidence of affective and cognitive involvement, derived from observational coding or validated engagement ratings
Motivation	Internal processes driving goal-directed behavior	Task persistence, preference ratings, caregiver/teacher reports	Willingness to continue; indirect motivational cues	Free-choice indices and voluntary re-engagement under optional access conditions, complemented by effort-based measures such as willingness to sustain effort as task demands increase
Sustained attention	Ability to maintain focus on a task over time	Looking time, reduced distractibility, on-task behavior	Attentional stability and focus maintenance	Performance-based indices over time, such as variability and decrement across blocks, complemented by eye-tracking metrics of attentional stability, including fixation stability and dispersion patterns

**Figure 1:**
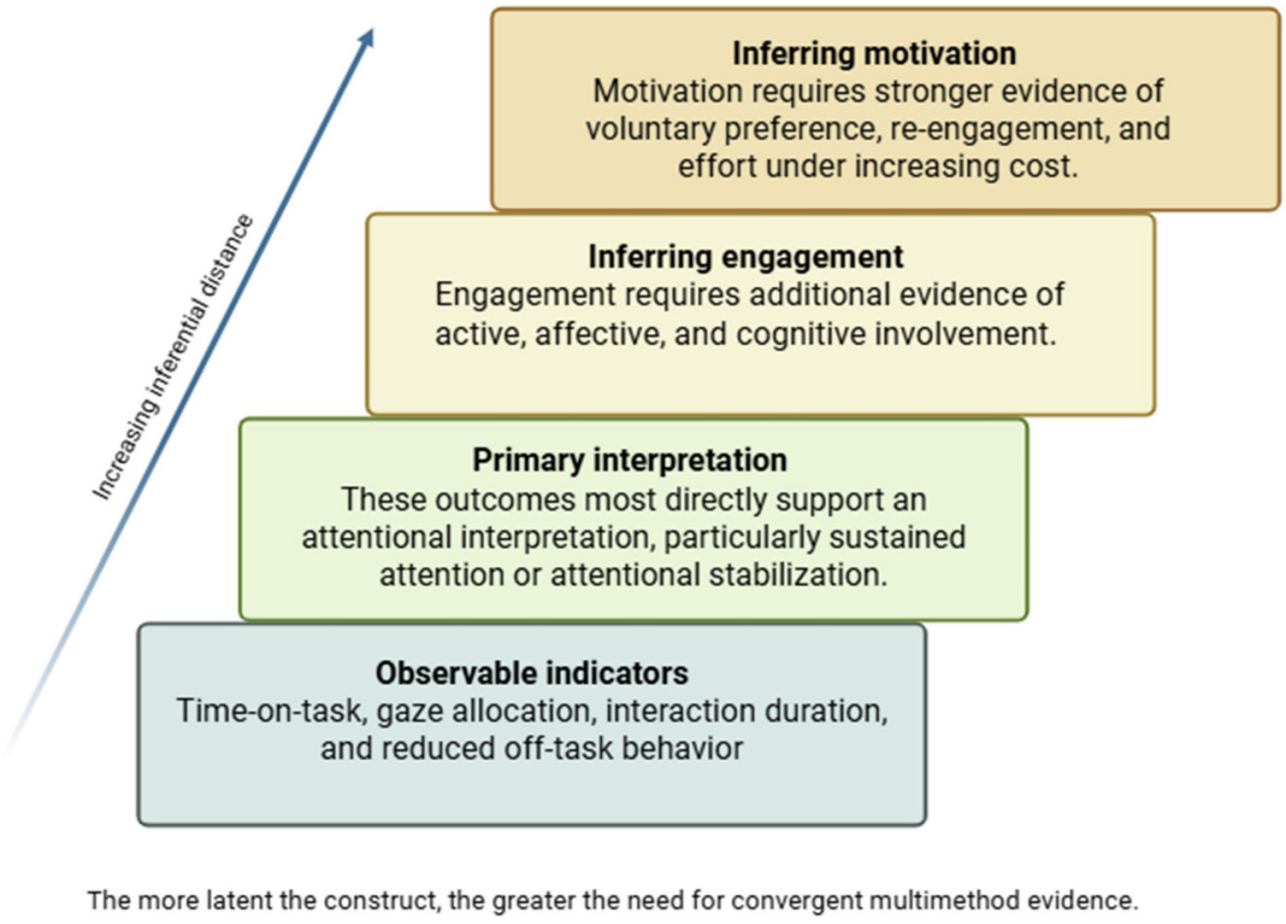
Inferential ladder for interpreting technology-related effects in ASD. Commonly reported behavioral indicators such as time-on-task, gaze allocation, interaction duration, and reduced off-task behavior most directly support an attentional interpretation. Stronger inferences about engagement and motivation require additional convergent evidence.

To complement Table 1, Figure 1 summarizes the inferential logic underlying the present argument, showing how commonly reported behavioral indicators most directly support an attentional interpretation, whereas stronger inferences about engagement and motivation require progressively more convergent evidence.

This framework helps clarify why many technology-related outcomes reported across domains may be interpreted more parsimoniously as markers of sustained attention, unless supported by additional construct-specific measures.

## 3 What existing studies actually show about technology-related outcomes

Across research domains involving social robots, virtual and augmented reality, and ICT-based educational tools, outcomes often reported in relation to technology use concern stability of focus, reduced distractibility, and longer periods of continuous task involvement. Although these outcomes are often discussed under the umbrella of “engagement,” they most directly reflect behavioral markers of sustained attention, and do not, on their own, allow robust inferences about engagement or motivation. In studies employing social robots, improvements are typically quantified through observable behavioral indices such as duration of interaction, number of responses, gaze or attentional focus, and reductions in off-task behavior (18, 19). These parameters, while informative, capture attentional allocation and task adherence more than they index intentional involvement or intrinsic motivation. Moreover, many of these investigations rely on small samples and short interaction periods, limiting the ability to determine whether these patterns persist over time or generalize beyond the robotic setting. A similar pattern emerges in research on immersive technologies. Virtual and augmented reality studies frequently report maintained visual focus, high task adherence, and lower behavioral variability during exposure (15). These findings indicate that structured and perceptually coherent environments can effectively support attentional stability. However, few studies incorporate measures capable of distinguishing attentional maintenance from deeper forms of emotional or cognitive engagement. The brief duration of most VR sessions further constrains the interpretation of whether these attentional effects are driven by novelty, by task design, or by more stable behavioral tendencies. ICT tools used in educational or home-based contexts show a comparable evidence pattern. Increases in participation, engagement, or motivation are often inferred from teacher reports, caregiver feedback, or other non-standardized indicators (8, 17). In these contexts, higher on-task behavior may be attributable to the alignment between the technological tool's characteristics such as immediate feedback, repetitiveness, or visual clarity and attentional preferences frequently observed in autistic learners. Only a minority of studies go beyond immediate behavioral indicators by employing cognitive tasks, objective engagement measures, or longitudinal designs that could help disentangle motivational contributions from attentional ones (20, 21). Notably, across these domains, engagement is rarely operationalized independently of attentional or time-based indicators. Taken together, current findings indicate that technology-related outcomes are most consistently captured through behavioral indicators of attentional stability during task performance. This does not imply that engagement is absent, nor that attention and engagement are mutually exclusive. Rather, sustained attention may scaffold participation and, in some contexts, contribute to engagement, but the indicators most commonly reported are insufficient to determine when such broader engagement is present. This does not diminish the potential usefulness of technological tools; rather, it highlights the need to specify which psychological processes are being modulated. Importantly, this interpretation should not be taken to suggest that technology-related effects are reducible to sustained attention in all cases. In some contexts, stronger inferences about engagement or motivation may be warranted, particularly when individuals show active participation, voluntary return to the activity, preference under free-choice conditions, or persistence beyond the initial exposure period. Such patterns would be more difficult to explain solely in terms of attentional capture or task structure and may instead point to broader forms of involvement. The critical issue, therefore, is not to replace one global interpretation with another, but to determine which construct is most strongly supported by the available evidence in each case. Without distinguishing attentional from motivational mechanisms, the interpretation of these behavioral changes remains closely tied to the type of indicators used to capture technology-related outcomes.

## 4 An alternative interpretation: technology as a modulator of sustained attention

The pattern synthesized here suggests that many behavioral changes observed during technology use may primarily reflect modulations of sustained attention and, when considered in isolation, do not provide sufficient evidence to conclude that engagement or motivation has increased. This interpretation does not negate existing explanations; instead, it offers a complementary perspective that may clarify the nature of the effects reported. Technological tools commonly used in autism interventions social robots, immersive environments, and digital applications often incorporate features that can naturally support attentional maintenance: predictable structure, reduced social ambiguity, immediate and consistent feedback, controlled repetition, and visual coherence. These characteristics align with perceptual preferences and processing styles frequently documented in autistic individuals, who may show stable responses to structured, orderly, and easily anticipable stimuli. In this sense, technology may function as a context that facilitates attentional stability, while the available indicators often do not allow emotional or motivational involvement to be disentangled from attentional maintenance. From a neurocognitive perspective, sustained attention is a dynamic and resource-limited process that fluctuates over time and is modulated by arousal, cognitive control, and motivational factors, rather than a stable state (22, 23). A further consideration involves the possible role of restricted interests. In some cases, digital tools incorporate visual, thematic, or procedural elements that resonate with circumscribed interests or preferred patterns common in autism. This correspondence may enhance attentional persistence during interaction without allowing a clear inference about intrinsic motivation toward the task itself. From this perspective, behaviors interpreted as “high engagement” may emerge from the alignment between stimulus characteristics and the individual's cognitive–attentional style. Recognizing this possibility does not diminish the value of technology; rather, it emphasizes that similar observable behaviors can arise from different underlying processes. If what is labeled as “engagement” is substantially driven by attentional support, understanding this relationship becomes essential. Such an understanding would allow for more precise interpretation of study outcomes, more targeted design of technological tools aligned with autistic cognition, and clearer identification of which components of the technology are most effective in supporting attention, which in fostering motivation, and which in promoting active participation. Importantly, engagement is typically inferred from observable behavior, whereas sustained attention reflects a cognitive process that can be indexed more directly through time-based and performance-related measures.

## 5 Discussion

This Perspective proposes an alternative interpretive framework for understanding technology-related outcomes in autism by shifting the focus from observable involvement to the cognitive processes that may be supported during digital interaction. Notably, outcomes often labeled as “engagement” are frequently derived from behavioral proxies such as gaze, interaction duration, time-on-task, or reductions in off-task behavior that may, in many cases, more directly reflect sustained attentional stability. As a result, the interpretation of technology-related effects appears to depend critically on how outcomes are defined and operationalized (11, 15). Making the distinction between observable indicators and inferential constructs explicit may therefore reduce the risk of conceptual overextension and strengthen the validity of inferences drawn from available measures.

Within this perspective, a key implication for the evaluation of technology-based interventions concerns the alignment between the hypothesized target process and the selected outcome measures. When the intended target is motivational engagement or intentional participation, purely time-based indicators, considered in isolation, may not be sufficiently specific to support strong inferences about multidimensional constructs (11, 13). By contrast, if sustained attention is a plausible mechanism, the same indicators may be appropriate provided they are interpreted as markers of attentional maintenance rather than direct evidence of motivation (14). Accordingly, more transparent reporting clarifying whether “engagement” refers to behavioral participation, cognitive investment, affective involvement, or attentional maintenance may improve comparability across studies and clarify what a given technology is most likely supporting (13).

In this context, complementary measures may strengthen construct-level inferences, provided that they are not treated in isolation as direct proxies of motivation or engagement. Performance-based indices over time, eye-tracking measures of attentional stability, and, where feasible, physiological or neurocognitive markers such as autonomic indices, pupillometry, or EEG may help clarify whether a given technology-related effect primarily reflects attentional maintenance, arousal regulation, or broader forms of involvement (24). Likewise, free-choice paradigms, voluntary re-engagement under optional access conditions, and effort-based tasks may offer more specific evidence when stronger claims about motivational processes are intended (25, 26). Their contribution lies less in any presumed specificity when considered alone than in their convergence with behavioral, observational, and standardized assessments.

These considerations also bear directly on the design, evaluation, and reporting of technology-based interventions in both research and clinical settings. When the intended target is attentional support, time-based and on-task indicators may be appropriate, provided that they are interpreted accordingly. When the aim is instead to support engagement, motivation, or active participation, stronger claims should be accompanied by construct-aligned measures capable of capturing these processes more directly. Greater precision in outcome reporting may also help clinicians and educators determine whether a given tool is primarily supporting attentional stability, facilitating task participation, or fostering broader forms of involvement, thereby improving both intervention design and the interpretation of technology-related effects across contexts.

Looking ahead, future research may benefit from study designs and measurement strategies that more clearly dissociate attention, motivation, and participation. For instance, multi-method approaches could combine behavioral metrics with standardized instruments and, where feasible, complementary measures that do not collapse into time-on-task alone. In addition, repeated-session protocols and follow-up assessments may help test temporal stability and clarify the extent to which observed patterns depend on novelty or highly structured settings, consistent with sustained attention as a dynamic and resource-limited process (22, 23). Finally, systematically manipulating common design features such as predictability, sensory coherence, feedback structure, and cognitive load may help identify which components primarily support attentional maintenance and which are more plausibly associated with deeper forms of engagement and intentional participation, particularly in educational contexts where engagement, participation, or motivation are often inferred from teacher reports, caregiver feedback, or other non-standardized indicators (8, 17). Overall, greater conceptual specificity and improved outcome operationalization may represent an important step toward more reliable interpretation of technology-related effects in autism and, in turn, guide the development of digital interventions that are more targeted, interpretable, and scalable.

### 5.1 Toward a testable research agenda

Future work should move beyond asking whether technology simply “enhances engagement” in autism and instead address a more fundamental question: which psychological processes are being supported, under what conditions, and on the basis of which evidence. This shift opens a set of more precise questions for the field. Do increases in time-on-task persist once novelty declines, or are they primarily driven by initial attentional capture? Which design features of technological tools preferentially support attentional stabilization, and which are more likely to foster active participation or voluntary re-engagement? When does apparent preference for a technological activity reflect genuine motivation rather than the effects of predictability, reduced ambiguity, or structured task demands? And how do these processes vary across autistic individuals with different cognitive, sensory, and communicative profiles? Framed in this way, future research can move beyond generic claims about “engagement” toward a more precise, testable, and mechanistically informed account of how technology-related outcomes should be interpreted in autism.

## Data Availability

The original contributions presented in the study are included in the article/Supplementary Material, further inquiries can be directed to the corresponding author.
