# Supplementary material for: Engagement, motivation, or sustained attention? Rethinking the effects of technology in autism
Source: Front Digit Health. 2026 Apr 17;8:1802286. doi: 10.3389/fdgth.2026.1802286 (PMC13132831; doi:10.3389/fdgth.2026.1802286)
Supplement: Supplementary file 1 [file Table1.docx]

**Supplementary Table S1. Descriptive mapping of illustrative studies included to exemplify recurrent measurement practices in technology-based autism research.** The table summarizes selected studies across robotics, virtual/augmented reality, and ICT-based interventions, indicating the construct labels used by the original authors, the primary indicators adopted, the presence of convergent measures, and the type of inference most directly supported.

| **Study** | **Technology domain** | **Target/context** | **Construct label used by original authors** | **Primary indicators used** | **Convergent measures included?** | **Type of convergent measure** | **Inference most directly supported** | **Interpretive note** |
| --- | --- | --- | --- | --- | --- | --- | --- | --- |
| Al-Nafjan et al., 2023 | Social robotics / robot-assisted therapy | Clinical therapy setting; NAO-based interaction with autistic children | Engagement | Video-based facial/head/eye detection; facial landmark extraction; CNN classification of frames as “engaged” vs “not engaged”; engagement percentage | Yes | Therapist-completed behavior-based assessment sheet; agreement with model output | Mixed attentional/engagement | Although the study includes therapist validation, engagement is still inferred primarily from observable behavioral and affective cues during brief robot-assisted interactions, rather than from direct evidence of motivation. |
| Tarantino et al., 2023 | VR/AR / immersive applications | Evaluation of engagement and appropriateness of two headsets (Oculus Rift and HoloLens) in high-functioning autistic young adults | Engagement | Behavioral observation measures: suspension of disbelief, body participation, exploration, action, facial expression, level of attention, emotional participation, verbal reaction. | Yes | Two complementary behavioral-observation frameworks (IVE-specific and generic engagement factors), plus post-session feedback/interview | Mixed attentional/engagement | The study broadens engagement assessment beyond simple time-based indicators, but the inference remains grounded mainly in observed behavioral involvement and attentional allocation rather than in direct motivational evidence. |
| Lyu et al., 2024 | AR / AI-supported educational tool | Tablet-based AR game for social-emotional learning with caregiver participation in autistic children | Engagement; attention; motivation-related involvement | User experience/usability outcomes; observed attention and sustained engagement; learning performance | Yes | Controlled comparison with baseline; quantitative and qualitative evaluation; caregiver participation; learning outcomes | Mixed attentional/engagement | The study emphasizes engagement within an educational AR context, but the reported evidence primarily reflects usability, attention, and task-related involvement rather than a clear dissociation between attentional and motivational processes. |
| Kumazaki et al., 2019 | Social robotics / digital agents | Preschool social-bid task comparing android robot, simple humanoid robot, digital avatar, and human in children with ASD vs TD | Social attention; preference / motivation to socially relevant behaviors | Percentage of head turns / gaze orientation toward the agent following a social bid; response rates across agents | Limited | Group comparison with TD controls; correlations with age, SRS-2, SCQ, and CDI | Primarily attentional | The paper discusses the potential of technological agents to “increase motivation to socially relevant behaviors,” but the actual outcome measure is response orienting to social bids, which more directly indexes social attention and attentional allocation than engagement or intrinsic motivation. |
| García-Martínez et al., 2025 | Social robotics / human–robot interaction | Turn-taking game with a social robot; RJA active vs inactive | Engagement | Head-pose telemetry; distraction/focus during interaction | Yes | UES-SF self-report questionnaire + telemetry-based attentional metrics | Mixed attentional/engagement | This study provides a stronger convergent assessment by combining telemetry-based attentional metrics with self-reported engagement, although it still does not include direct measures of motivation. |
| Koomaran & Sani, 2025 | Gamified educational tools / ICT-based learning | Classroom gamification for students with ASD; qualitative teacher perspectives | Motivation; engagement | Teacher-reported focus, participation, excitement, interaction, communication, collaboration, response to rewards/feedback | No | Semi-structured teacher interviews; thematic analysis | Mixed attentional/engagement | Motivation and engagement are inferred mainly from teacher-reported participation, focus, and classroom involvement, without standardized or convergent measures capable of distinguishing attentional from motivational contributions. |
| Ribeiro Silva et al., 2025 | Gamified educational technology / ICT-based learning | Gamified educational technology for children with autism; single-subject design | Engagement; learning gains | Correct answers, undesired behaviors, ABA-related skills, positive behaviors | Limited | Single-subject behavioral assessment | Mixed attentional/engagement | Engagement is discussed explicitly, but the available indicators are primarily behavioral and performance-based, supporting a mixed attentional/engagement interpretation rather than a direct motivational inference. |
| Carnahan et al., 2009 | Low-tech educational intervention / classroom instructional design | Small-group literacy instruction for students with autism and significant learning needs | Engagement | Interval-coded on-task engagement; body/face/eyes oriented toward materials; appropriate use of materials; relevant verbalizations; absence of unrelated repetitive vocalizations | Limited | Teacher social-validity survey; ABCAC single-subject design | Primarily attentional | Engagement is operationalized through observable on-task behavior and orientation toward instructional materials, providing limited evidence for stronger inferences about motivation or deeper forms of involvement. |
